# Intracellular phosphate recycling systems for survival during phosphate starvation in plants

**DOI:** 10.3389/fpls.2022.1088211

**Published:** 2023-01-17

**Authors:** Yushi Yoshitake, Kohki Yoshimoto

**Affiliations:** Department of Life Sciences, School of Agriculture, Meiji University, Kawasaki, Japan

**Keywords:** phosphate recycling, phosphatase, nuclease, membrane lipid remodeling, autophagy, vacuolar transporter

## Abstract

Phosphorus (P) is an essential nutrient for plant growth and plants use inorganic phosphate (Pi) as their P source, but its bioavailable form, orthophosphate, is often limited in soils. Hence, plants have several mechanisms for adaptation to Pi starvation. One of the most common response strategies is “Pi recycling” in which catabolic enzymes degrade intracellular constituents, such as phosphoesters, nucleic acids and glycerophospholipids to salvage Pi. Recently, several other intracellular degradation systems have been discovered that salvage Pi from organelles. Also, one of sphingolipids has recently been identified as a degradation target for Pi recycling. So, in this mini-review we summarize the current state of knowledge, including research findings, about the targets and degradation processes for Pi recycling under Pi starvation, in order to further our knowledge of the whole mechanism of Pi recycling.

## Introduction

**Figure 1:**
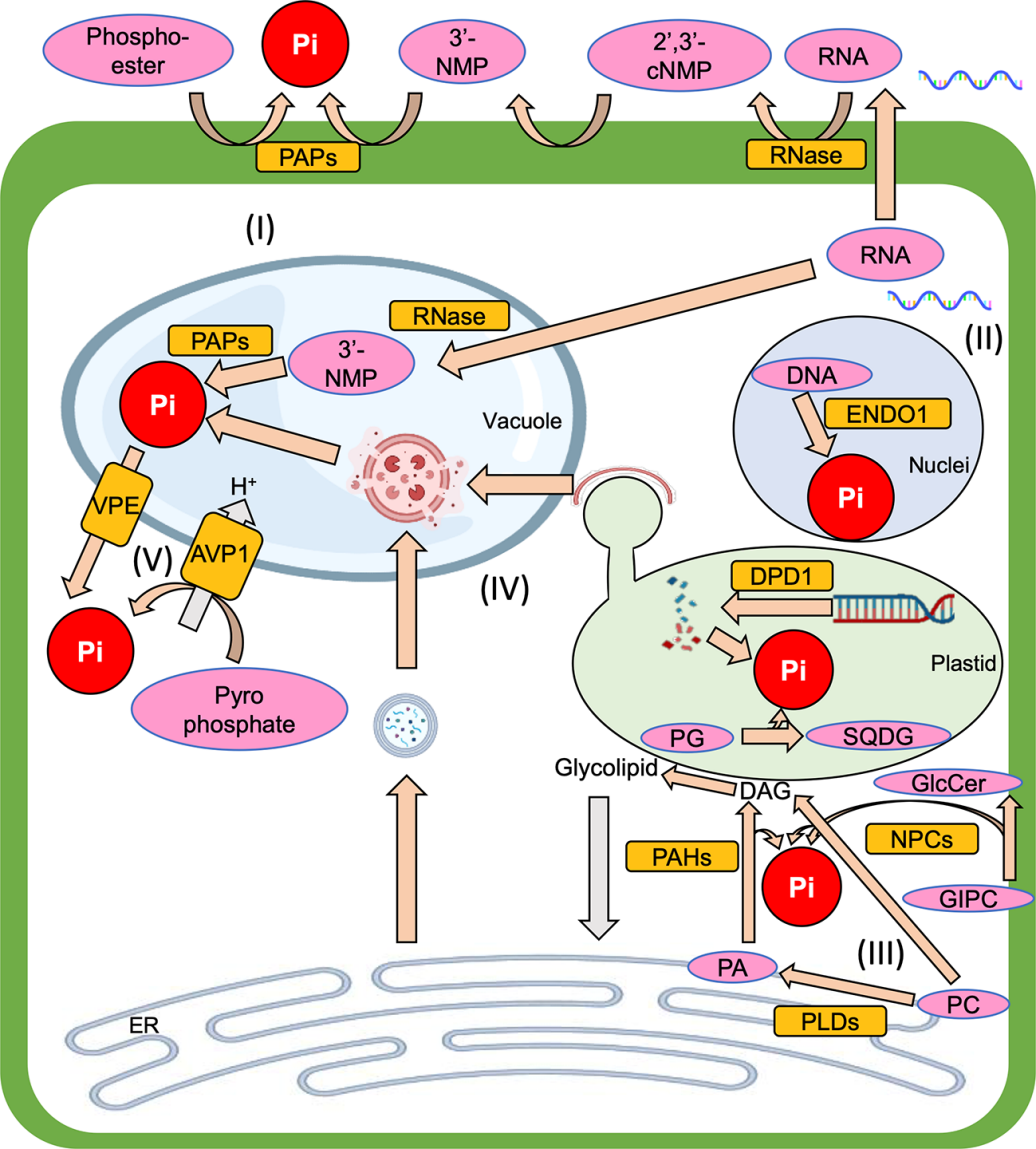
The overview of Pi recycling systems which are introduced in this mini-review, (I) Phosphoester degradation, (II) Nucleic acid degradation, (III) Membrane lipid remodeling, (IV) Autophagy, (V) Vacuolar transporter. Pi, inorganic phosphate; PAP, purple acid phosphatase; RNase, Ribonuclease; 2’,3’-cNMP, 2’,3’-cyclic nucleoside monophosphate intermediate; 3’-NMP, 3’-nucleoside monophosphate; ENDO1, endonuclease 1; DPD1, defective in pollen organelle DNA degradation 1; PG, phosphatidylglycerol; SQDG, sulfoquinovosyldiacylglycerol; GIPC, glycosylinositolphosphorylceramide; GlcCer, glucosylceramide; ER, endoplasmic reticulum; PC, phosphatidylcholine; PA, phosphatidic acid; DAG, diacylglycerol; PLC, phospholipase C; NPC, non-specific PLC; PLD, phospholipase D; PAH, phosphatidic acid phosphohydrolase; VPE, vacuolar pi efflux transporter; AVP1, arabidopsis vacuolar pyrophosphatase 1.

Plants take up inorganic phosphate (Pi; PO_4_^3-^) and use it as their phosphorus (P) source. In the plant, the Pi is metabolized into organic phosphate, and it is also used for post-translational protein modification to regulate protein activity. Thus, Pi is essential for proper plant growth. Since Pi makes easily complex with metal ions such as aluminum and iron ions in soils and thus render it unavailable to plants, however, soils with low orthophosphate, which is an available form of Pi, are widespread throughout the world (Raghothama, 1999; Lynch, 2011). So, to overcome Pi starvation stress, plants have various systems to respond to Pi starvation. One of the Pi starvation responses is “Pi recycling”, which is a complex subject due to the existence of many intracellular components that contain Pi and the many degradation systems that can salvage Pi from them (**Figure 1**). Therefore, this mini-review aims to summarize our current understanding of Pi recycling in plant cells under Pi starvation and highlight areas that require further study to develop a better understanding of the overall Pi recycling process.

## Phosphoester degradation

Pi starvation-induced acid phosphatases PURPLE ACID PHOSPHATASEs (PAPs), which have phosphoester hydrolase activity, have been purified and characterized in many plant species, such as *Arabidopsis thaliana* (Li et al., 2002), *Solanum lycopersicum* (Bozzo et al., 2002; Bozzo et al., 2006; Srivastava et al., 2020), *Nicotiana banthaminana* (Lung et al., 2008), *Lupinus albus* (Ozawa et al., 1995; Li and Tadano, 1996; Miller et al., 2001; Aslam et al., 2022). It was reported that the expressions of some *PAP* genes are upregulated by Pi starvation in *A. thaliana* and *S*. *lycopersicum* (del Pozo et al., 1999; Li et al., 2002; Wang et al., 2011; Srivastava et al., 2020). Of these, AtPAP10, 12, 17, and 26 are secreted acid phosphatases (Veljanovski et al., 2006; Hurley et al., 2010; Tran et al., 2010; Wang et al., 2014; O’Gallagher et al., 2022), whereas, AtPAP12, 15, and 16 are known to be major intracellular acid phosphatases in *A. thaliana*, because intracellular acid phosphatase activity in *pap12*, *pap15*, and *pap26* is less than that in wild-type (Wang et al., 2014). Additionally, since AtPAP15 is not expressed in the root hairs or epidermal cells, it is unlikely to be secreted into the root exudates (Kuang et al., 2009). Recently, it was also reported that under Pi starvation AtPAP17 localizes in the lytic vacuole, in addition to extracellular spaces (O’Gallagher et al., 2022). AtPAP17 and 26 are bifunctional enzymes, both phosphatase and peroxidase (del Pozo et al., 1999; Veljanovski et al., 2006). Since it is known that Pi starvation enhances accumulation of reactive oxygen species (ROS), which leads to programmed cell death (Van Camp et al., 1998; Overmyer et al., 2000; Tyburski et al., 2009; Yoshitake et al., 2022), that proteins may contribute to plant growth not only through the resupply of Pi from intracellular components but also because increase ROS degradation under Pi starvation. It is known that a phosphohydrolase for inositol pyrophosphates (PP-InsPs), one of the phosphoesters, has a role in Pi starvation response, but it is unclear whether this enzyme is involved in Pi recycling (Gaugler et al., 2022).

## Nucleic acid degradation

Pi can be salvaged from degradation of nucleic acids under Pi starvation. Nucleic acids contain Pi, and most nucleic acid in plant cells is ribosomal RNA (rRNA) (Veneklaas et al., 2012). They are broken down by Ribonucleases (RNases). The T2 family of RNases are highly conserved among viruses, bacteria, fungi, animals, and plants (Deshpande and Shankar, 2002), and can be grouped into three subclasses (Igic and Kohn, 2001). Expression of class I RNases is induced by stresses such as wounding, drought, and salinity (Hillwig et al., 2008; Bustos et al., 2010; MacIntosh et al., 2010), and also by Pi starvation (Bariola et al., 1994; Köck et al., 1995; Dodds et al., 1996; Bariola et al., 1999). These facts, plus the following facts lead us to hypothesize that these RNases degrade apoplastic RNA and salvage Pi from apoplast under Pi starvation. First, most class I RNases are secreted to out of the cell (Köck et al., 1995; Bariola et al., 1999; Smith et al., 2018). Also, Arabidopsis apoplastic fluid contains small RNAs and long noncoding RNAs, including circular RNAs (Zand Karimi et al., 2022). Furthermore, exogenous addition of RNA in media recovers growth defect of primary roots under Pi starvation (Chen et al., 2000). It is also thought that carnivorous plants, *Drosera aldelae* and *Nepenthes ventricose*, salvage Pi from the degradation of prey RNA by RNase (Okabe et al., 2005; Stephenson and Hogan, 2006).

Furthermore, it is thought that class I RNase can salvage intracellular Pi during Pi starvation. For example, in *S*. *lycopersicum*, RNase LX is a Pi starvation-induced intracellular RNase (Köck et al., 1995), which is retained in endoplasmic reticulum (ER) by C-terminal tetrapeptide HDEF (Löffler et al., 1993; Lehmann et al., 2001). Class II RNase also appear to be involved in response to Pi starvation. For example, Arabidopsis class II RNase, RNS2, is localized in the vacuole and degrade vacuolar RNA during development (Hillwig et al., 2011; Floyd et al., 2017). In addition, because the expressions of class II RNase genes are upregulated by Pi starvation, it is considered that these RNases have not only a housekeeping role in rRNA degradation but also a role in response to Pi starvation (Taylor et al., 1993; Liang et al., 2002; MacIntosh et al., 2010; Hillwig et al., 2011; Floyd et al., 2017).

Recently, it has been reported that AtRNSs can cleavage transfer RNAs (tRNAs) and that RNS1 is responsible for the accumulation of specific tRNA fragments (glycine and aspartic acid) in leaves under Pi starvation (Megel et al., 2019). However, it is still unclear whether the generation of tRNA fragments is involved in Pi recycling.

Pi is also released from 3’-nucleoside monophosphate (3’-NMP) by PAP (Abel et al., 2000). 3’-NMP can be generated from 2’,3’-cyclic nucleoside monophosphate intermediate (2’,3’-cNMP), which is generated from degradation of RNAs, by a side reaction of class I and II RNases (Nürnberger et al., 1990). However, the catabolic enzyme of 2’,3’-cNMP, a Pi starvation-induced cyclic nucleotide phosphodiesterase, was discovered in cultured *L*. *esculentum* cells, and it had higher activity of 2’,3’-cNMP degradation was higher than RNases (Abel et al., 2000). Thus, it appears that Pi salvaging from cyclic nucleotides is mainly mediated by this cyclic nucleotide phosphodiesterase pathway.

DNA also can be source of Pi. The *ENDONUCLEASE 1* (*ENDO1*) gene encodes a type I nuclease and its expression in Petunia is induced by Pi starvation (Pérez-Amador et al., 2000; Jones et al., 2021). Under Pi starvation, plastid DNA is degraded by DEFECTIVE IN POLLEN ORGANELLE DNA DEGRADATION 1 (DPD1), exonuclease. However, Pi remobilization from old leaves to younger leaves under Pi starvation is suppressed in the *dpd1* mutant (Takami et al., 2018), suggesting that DPD1 may be required for Pi recycling. However, it is unclear how the metabolites of plastid DNA are transported out of plastids.

## Membrane lipid remodeling

The major components of the biological membrane are glycerolipids, sphingolipids, and sterols (Harayama and Riezman, 2018). The glycerolipids containing Pi in the polar heads are called phospholipids, whereas, those containing carbohydrates are called glycolipids. Under Pi starvation, phospholipids are degraded and the resultant diacylglycerols (DAGs), which are precursors of glycolipids, are delivered to the plastid. The glycolipids are transferred to extraplastidic membranes such as the ER membrane, plasma membrane, and mitochondria membrane to maintain membrane structures (Härtel and Benning, 2000; Dörmann and Benning, 2002; Jouhet et al., 2004; Nakamura, 2013). This process is called “membrane lipid remodeling”. In this process, phosphatidylglycerol (PG) which is an anionic phospholipid, and phosphatidylcholine (PC) which forms a lipid bilayer, are replaced respectively by sulfoquinovosyldiacylglycerol (SQDG) and digalactosyldiacylglycerol (DGDG) (Härtel and Benning, 2000; Dörmann and Benning, 2002; Yu et al., 2002). PLASTID LIPASE 1 (PLIP1) and its paralogues (PLIP2, 3) degrade PG, but they produce fatty acids and lyso-PG rather than Pi (Wang et al., 2017; Wang et al., 2018). So, for full understanding of the Pi recycling system from PG, it is still necessary to identify the PG catabolic enzymes which produce Pi from the polar head of PG. SQDG is synthesized by SQDG SYNTHASE 2 (SQD2) from DAG and UDP-sulfoquinovose (Yu et al., 2002), with the UDP-sufoquinovose being generated by SQD1 (Sanda et al., 2001). The expression of both *SQD1* and *2* are upregulated by Pi limitation (Essigmann et al., 1998; Yu et al., 2002). SQD2 also synthesizes glucuronosyldiacylglycerol (GlcADG) and *sqd2* mutant showed more severe growth defect phenotypes than *sqd1* mutant under Pi starvation (Okazaki et al., 2013). Therefore, it appears that GlcADG has an important role in Pi starvation tolerance. However, it is not clear what lipids are replaced by GlcADG under Pi starvation.

There are two PC degradation pathways during membrane lipid remodeling. One pathway generates DAG from PC directly by phospholipase C (PLC), while the other generates DAG by phosphatidic acid phosphatase from phosphatidic acid (PA) which is produced from PC by phospholipase D (PLD). In *A*. *thaliana*, the expression of two *NON-SPECIFIC PLC* (*NPC4*, *5*) genes is upregulated by Pi starvation. Since NPC4 and NPC5 localize to the plasma membrane and cytoplasm respectively, it is thought that the localization for NPC4 and NPC5 degradation of PC is in the plasma membrane and ER membrane, respectively (Nakamura et al., 2005; Gaude et al., 2008). PHOSPHATE STARVATION-INDUCED GENE 2 (PS2) and its homologue, PHOSPHOETHANOLAMINE/PHOSPHOCHOLINE PHOSPHATASE 1 (PECP1), salvage Pi from phosphocholine (Angkawijaya and Nakamura, 2017; Hanchi et al., 2018; Angkawijaya et al., 2019).

*A*. *thaliana* has 12 *PLD* genes, of which two *AtPLDζ* (*PLDζ1* and *PLDζ2*) gene expression is upregulated by Pi starvation. Furthermore, *pldζ1 pldζ2* mutant has a defect in membrane lipid remodeling (Zhang et al., 2005; Li et al., 2006a; Li et al., 2006b; Su et al., 2018). PHOSPHATIDIC ACID PHOSPHOHYDROLASE 1, 2 (PAH1, PAH2) are soluble phosphatidic acid phosphatases and are localized in the cytoplasm (Nakamura et al., 2009). Under Pi starvation, the *pah1 pah2* double mutant shows growth defect phenotypes and its membrane lipid remodeling is suppressed, indicating that PAHs are important in the Pi recycling from phospholipids (Nakamura et al., 2009). It has also been found that while the growth phenotype of *pah1* and *pah2* single mutants was similar to that of wild-type, the *pah1 pah2* double mutant showed abnormal phenotypes, such as stutter rosette leaves, shorter siliques, and phospholipid accumulation, even under Pi-sufficient conditions (Eastmond et al., 2010). Therefore, it appears that PAH1 and PAH2 are functionally redundant. The more moderate phenotype of *pldζ1 pldζ2* double mutant compared with the *pah1 pah2* suggests that other PLDs provide PA to PAH for membrane lipid remodeling (Li et al., 2006b; Nakamura et al., 2009). After DAGs are generated by NPC or PAH, they are delivered to plastids by SECOND LptD-FAMILY PROTEIN (LPTD1) under Pi starvation (Hsueh et al., 2017). Then, the DAGs are converted into glycolipids on the plastid envelope membrane. MONOGALACTOSYLDIACYLGLYCEROL SYNTHASE 1 (MGD1), Type-A monogalactosyldiacylglycerol (MGDG) synthase, localizes on the inner envelope membrane of plastids and works in photosynthetic tissues even under Pi sufficient conditions (Awai et al., 2001; Kobayashi et al., 2007). In contrast, Type-B MGD (MGD2, 3) localizes on the outer envelope membranes of plastids, and the expression of these genes is strongly activated by Pi starvation (Awai et al., 2001; Kobayashi et al., 2004). MGDG, which is produced by Type-B MGD, is the substrate of DGDG SYNTHASE (DGD) that is transported to extraplastidic membranes to replace PC (Kelly et al., 2003). However, it is unclear how DGDG is delivered to extraplastidic membranes.

Recently, it has been reported that under Pi starvation the Pi-containing sphingolipid glycosylinositolphosphorylceramide (GIPC) is degraded by NPC4 whereas the amount of glucosylceramide (GlcCer), a non-Pi-containing sphingolipid, is increased (Yang et al., 2021). This finding suggests that GIPC is a Pi store and is replaced by GlcCer or metabolites from GlcCer, to maintain membrane functions. Therefore, both glycerophospholipids and phosphosphingolipids could facilitate Pi storage under Pi starvation.

## Autophagy

(Macro)autophagy is an evolutionally conserved process for degradation of protein and/or organelles in eukaryotes. In this process, targets are engulfed by an isolation membrane, forming an autophagosome. Then, the autophagosomes are delivered to the vacuole, where the targets are degraded. Plant autophagy is induced by nutrient starvation, such as carbon, nitrogen, and zinc deficiency (Yoshimoto et al., 2004; Izumi et al., 2010; Shinozaki et al., 2020). Pi starvation also induces autophagy in tobacco BY-2 cells and *A. thaliana* (Tasaki et al., 2014; Naumann et al., 2019; Yoshitake et al., 2022). Also, experiments using an ER stress sensor mutant have shown that Pi starvation-mediated autophagy is regulated by ER stress response (Naumann et al., 2019). ER stress induces ER-phagy, a type of autophagy that degrades ER specificity (Liu et al., 2012). Recently, it has also been reported that ER stress is induced by oxidative lipid accumulation and that ER-phagy contributes to Pi recycling in leaf cells (Yoshitake et al., 2022). Furthermore, the timing of inducing the ER-phagy mediated Pi recycling system is earlier than that of PHOSPHATE STARVATION RESPONSE 1 transcription factor regulated Pi starvation responses, such as membrane lipid remodeling (Pant et al., 2015), suggesting that plants have two phases in their Pi starvation response, to effectively adapt to natural fluctuations in Pi starvation.

Another kind of autophagy, chlorophagy, which involves specific chloroplast degradation by autophagy, also contributes to Pi recycling. For example, under Pi starvation, excess supply of nitrate induces Rubisco-containing body (RCB)-mediated chlorophagy, in which the chloroplast is partially degraded. This chlorophagy is induced by a reduction in the carbon/nitrogen ratio and is conducive to Pi recycling (Yoshitake et al., 2021). Therefore, in addition to the Pi recycling system that is induced by sole Pi starvation, plants seem to have another one which is induced by complex nutrient status.

Ribophagy is the process of ribosome degradation by autophagy, and it has been discovered in plants as well as in yeast (Kraft et al., 2008). RNA-containing granules have been shown to be incorporated into vacuoles of *Zea mays* primary root meristem (Niki et al., 2014). Also, a study on *A*. *thaliana* reported that the number of autophagosomes containing ribosomes was increased by defective of RNS2 (Floyd et al., 2015). Therefore, autophagy may play a role in RNA and ribosome degradation, although it is unclear whether this contributes to Pi recycling.

## Vacuolar transporter

The vacuole is a compartment for degradation of intracellular components and salvaging nutrients. Therefore, knowledge of the Pi transporters on the vacuolar membrane (tonoplast) is important for understanding the system of Pi homeostasis and Pi recycling. In yeast, the VACUOLAR TRANSPORTER CHAPERONE complex (Vtc1-4) transports Pi from the cytoplasm into the vacuole and generates polyphosphates, a linear chain of anywhere between three to thousands of Pi units (Secco et al., 2012b). Polyphosphate can be hydrolased to Pi by PHOSPHATE METABOLISM 5 (Phm5) in the vacuole and it is transported from the vacuole to the cytoplasm by the vacuolar Pi transporter Pho91 (Ogawa et al., 2000; Hürlimann et al., 2007). Vtc2-4 and Pho91 have SIG1/Pho81/XPR1 (SPX) domain, and it is known that plants have Pi transporters containing the SPX domain, SPX-MAJOR FACILITATOR SUPERFAMILY (SPX-MFS) family proteins (Secco et al., 2012a; Secco et al., 2012b). In *A. thaliana*, there are three SPX-MFS proteins, PHOSPHATE TRANSPORTER 5 (PHT5;1, PHT5;2, and PHT5;3) (Liu et al., 2016). The expression of *PHT5;1*/*VPT1* is induced by high Pi conditions in order to maintain the Pi concentration in the cytoplasm (Liu et al., 2015). There are also three SPX-MFS (OsSPX-MFS1-3) proteins in *Oryza sativa* (Wang et al., 2015). OsSPX-MFS3 transports Pi from the vacuolar lumen to the cytoplasm, whereas OsSPX-MFS1 and 2 import Pi to the vacuole (Wang et al., 2015; Liu et al., 2016; Guo et al., 2022). Other VACUOLAR Pi EFFLUX TRANSPORTERs (VPEs) have been identified in *O*. *sativa*, and phylogenic analysis has revealed that these VPE proteins evolved from an ancient plasma membrane glycerol-3-phosphate transporter during terrestrial plant evolution (Xu et al., 2019). The expression of VPE genes is induced by Pi starvation when they control Pi homeostasis in plant cells (Ramaiah et al., 2011; Xu et al., 2019). Some transporter proteins on the tonoplast generate Pi. For example, it has been shown that the transcription and translation of ARABIDOPSIS VACUOLAR PYROPHOSPHATASE 1 (AVP1), type I H^+^-pyrophosphatase, are enhanced by Pi starvation (Yang et al., 2007), and that vacuolar pyrophosphatase generates two Pis from one pyrophosphate during transportation of H^+^ into the vacuole (Zhen et al., 1994; Maeshima, 2000). Furthermore, overexpression of *AVP1* improved plant growth under Pi starvation in *A. thaliana*, *S. lycopersicum*, and *O. sativa* (Yang et al., 2007; Heuer et al., 2016), and AVP1 overexpressors have been shown to exhibit enhanced rhizosphere acidification under Pi starvation which enhanced Pi uptake (Yang et al., 2007). However, it is still unclear whether this pyrophosphatase activity relates to Pi recycling.

## Conclusion

Pi recycling systems are the plant response mechanism to Pi starvation. Therefore, it is important to increase our understanding of this important process. So, we examine the catabolic pathways producing Pi from intracellular components under Pi starvation in this mini-review. We have shown that there are various plant responses to Pi starvation. For example, while Pi starvation induced the expression of *ENDO1* in Petunia, this did not occur in *A. thaliana* grown on Pi-depleted media for 14 days (Pérez-Amador et al., 2000; Jones et al., 2021). Also, recent studies using *A*. *thaliana* showed that ER-phagy only contributes to Pi recycling in the early phase of Pi starvation (2-3 days) and that RCB-mediated chlorophagy salvages Pi under high nitrate/Pi starvation conditions (Yoshitake et al., 2021; Yoshitake et al., 2022). Hence, AtENDO1 may be involved in Pi recycling in early phase of Pi starvation or under certain conditions.

In yeast, the VTC complex is required for microautophagy, another type of autophagy in which the tonoplast invaginates and directly engulfs the target (Uttenweiler et al., 2007). But, while microautophagy is known to be induced by photodamage, sucrose starvation, and ammonium stress (Nakamura and Izumi, 2018; Goto-Yamada et al., 2019; Robert et al., 2021), it is still unclear whether Pi starvation induces microautophagy. Also, it is possible that other intracellular degradation systems could be involved in Pi recycling, in addition to microautophagy.

Therefore, in conclusion, further analysis is still needed to fully understand the whole Pi recycling system. This includes more research under various Pi starvation conditions, and studies to establish the extent to which other degradation systems are involved in Pi recycling.

## Author contributions

YY and KY wrote the manuscript. All authors contributed to the article and approved the submitted version.
